# Determination of Optical Purity of Lactic Acid-Based Chiral Liquid Crystals and Corresponding Building Blocks by Chiral High-Performance Liquid Chromatography and Supercritical Fluid Chromatography

**DOI:** 10.3390/molecules24061099

**Published:** 2019-03-20

**Authors:** Anna Poryvai, Terezia Vojtylová-Jurkovičová, Michal Šmahel, Natalie Kolderová, Petra Tomášková, David Sýkora, Michal Kohout

**Affiliations:** 1 Department of Organic Chemistry, University of Chemistry and Technology Prague, Technická 5, 166 28 Prague, Czech Republic; poryvaia@vscht.cz (A.P.); smahelm@vscht.cz (M.Š.); natalie.kolderova@gmail.com (N.K.); 2 Institute of Physics, Academy of Sciences of the Czech Republic, Na Slovance 2, 182 21 Prague, Czech Republic; vojtyl@fzu.cz (T.V.-J.); tomasko@fzu.cz (P.T.); 3 Forensic Laboratory of Biologically Active Substances, University of Chemistry and Technology Prague, Technická 3, 166 28 Prague, Czech Republic; 4 Department of Analytical Chemistry, University of Chemistry and Technology Prague, Technická 5, 166 28 Prague, Czech Republic; sykorad@vscht.cz

**Keywords:** chiral liquid crystals, optical purity, chiral separation, supercritical fluid chromatography, enantioseparation of liquid crystals, mass spectrometry detection, mesomorphic properties

## Abstract

Liquid crystals (LCs) are among the most prominent materials of the current information age, mainly due to their well-known application in liquid crystal displays (LCDs). Their unique electro-optical properties stem from their ability to form organised structures (mesophases) on the transition from solid state to isotropic liquid. Molecules of LCs in a mesophase still maintain the anisotropy of solid crystals, while simultaneously exhibiting the fluidity of liquids, which gives the system the ability to react immediately to external stimuli such as electric or magnetic fields, light, mechanical stress, pressure and, of course, temperature. For the proper function of LC-based devices, not only chemical, but also optical purity of materials is strongly desirable, since any impurity could be detrimental to the self-assembly of the molecules. Therefore, in this study we aimed to verify synthetic methods published in the literature, which are used nowadays to prepare chiral building blocks based on lactic acid, for their enantioselectivity. Moreover, we have focused on the development of an analytical chiral separation method for target liquid crystalline materials. Using a chiral polysaccharide-based column operated in liquid chromatography mode, we show that not all published methods of LC synthesis are enantioselective, which could lead to significant differences in the properties of the resulting materials. We show that high-performance liquid chromatography with UV detection and supercritical fluid chromatography with UV and mass spectrometry detection enable full control over the chemical and optical purity of the target LCs and the corresponding chiral building blocks. For the first time, we utilise supercritical fluid chromatography with mass detection for the direct chiral analysis of liquid crystalline materials and impurities formed during the synthesis.

## 1. Introduction

Liquid crystals (LCs) represent one of the most prominent classes of materials of today’s information age. The molecules of LCs can self-assemble into organised supramolecular structures (mesophases) that combine the fluidity of liquids with the long-range order of solids [1,2]. Molecules in a mesophase readily respond to external stimuli such as electric and magnetic field, light and mechanical stress. Thanks to these properties, LCs have found many applications, for example, in liquid crystal displays (LCDs), optical shutters, light beam steering and shaping [3,4].

Among LCs, chiral liquid crystals (CLCs) represent an important class of materials that can self-assemble into chiral mesophases [5]. Macroscopic chirality of the mesophases results in special properties, such as (anti)ferroelectricity, selective reflection of light, and heat sensitivity, which render CLCs ideal candidates for the fabrication of high-speed high-contrast LCDs, photonic devices, and contact thermography devices, respectively [1,6,7]. Since the self-assembly of LCs is very sensitive to any impurities present in the bulk material, control of chemical and optical purity during the synthesis of target CLCs is essential. Any impurity may result in the alteration of the mesophase structure or even complete loss of mesomorphic behaviour.

The optical purity of CLCs was in the past mainly controlled by optical rotation measurement and transformation of precursors to diastereoisomers [8]. However, such transformation does not usually provide information on trace amounts of other stereoisomers, which can be present in the final material. Even a trace of the opposite enantiomer may consequently lead to variations in the mesomorphic behaviour expected for the enantiomerically pure material. The source of such optical impurity may be the starting material, which frequently contains a trace amount of the opposite enantiomer, or it may occur during synthesis due to partial racemisation of an intermediate upon its synthetic modification. Although the optical purity of CLCs is a very important issue in the development and utilisation of novel chiral materials, only scarce information on chiral separation of CLCs can be found in the literature [9,10]. Most of the work thus far has been devoted to chiral liquid chromatography separation of photosensitive CLCs and the effect of light-induced *E*-to-*Z*-isomerisation on chiral recognition [11,12,13]. It has been found that the structure of CLCs plays an important role in the chiral resolution. It has also been documented that even materials bearing very small substituents at the chiral centre can be efficiently separated on polysaccharide-based chiral columns [9,10]. However, to the best of our knowledge, there is no systematic study dealing with the control of enantioselectivity of the synthetic protocol leading to CLCs using contemporary analytical chemistry instrumentation.

Therefore, we have focused on controlling enantioselectivity of the synthesis of a novel type of lactic acid-based CLCs. We used three different synthetic procedures reported in the literature, which have frequently been used for the preparation of the chiral intermediates required for the synthesis of target lactic acid-based CLCs. We developed a method for the chiral resolution of both enantiomers of the chiral precursors using the synthesised enantiomers and verified the enantioselectivity of the synthetic pathways. Moreover, we elaborated a method for the analytical enantioseparation of the target liquid crystalline materials, not only in HPLC, but also in supercritical fluid chromatography (SFC) with mass spectrometry (MS) detection. We document that the small percentage of the opposite enantiomer present in commercially available chiral starting materials increases during the synthesis, thereby affording materials that are not optically pure. The described chiral separation procedure enables very precise control of the chemical and optical purity of the target CLCs, which is required for practical applications.

## 2. Results and Discussion

### 2.1. Synthesis of Chiral Building Blocks

**Figure 1:**
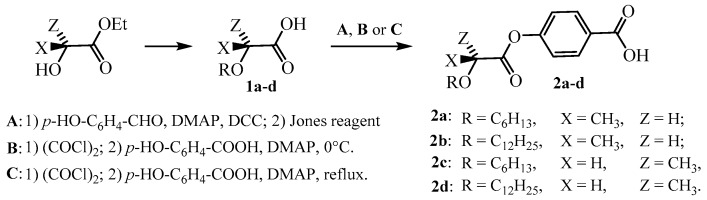
Synthesis and designation of the chiral precursors prepared by three different methods; (**A**) four step method using oxidation of an aldehyde as an intermediate step, (**B**) and (**C**) direct acylation of 4-hydroxybenzoic acid with the chiral acid under different conditions.

The studied chiral intermediates (Figure 1) were prepared using previously described synthetic procedures and characterised by nuclear magnetic resonance techniques to comply with the data published in the literature [8,14,15,16,17,18]. To clarify the differences among the three methods used here, the experimental procedures are described below in brief. The method (B) is a modified version of a synthetic procedure already described in the literature [17].

The first two steps [8,15,16] of the synthesis are common to all three procedures, and they were accomplished as follows: A suspension of silver(I) oxide, alkyl iodide and ethyl (*S*)-lactate (or methyl (*R*)-lactate) in diethyl ether was stirred for 90 h in the absence of UV light. Then, the reaction mixture was filtered, solvent was evaporated and the product (colourless liquid) was purified via distillation under reduced pressure (*p* = 0.6 Torr, t = 54–55 °C for *S*-C6; *p* = 2.4 Torr, t = 143–146 °C for *S*-C12). Subsequently, an aqueous solution of lithium hydroxide (1.3 M) was added dropwise to a stirred solution of ethyl *O*-alkyllactate in methanol (with THF in the case of dodecyl derivative) at 0 °C. The reaction mixture was stirred for 3 days at room temperature, then it was acidified (pH = 2) with 17% aq. hydrochloric acid and extracted with dichloromethane (3 × 20 mL). The combined organic solution was dried with anhydrous magnesium sulphate. Solvent was removed under reduced pressure and the corresponding *O*-alkyllactic acid was isolated as a colourless liquid. For analytical data of the derivatives, see ESI.

#### 2.1.1. Method (A) 

To the solution of **1a** in dry DCM, *p*-hydroxybenzaldehyde, *N*,*N*’-dicyclohexylcarbodiimide (DCC) and 4-*N*,*N*-dimethylaminopyridine (DMAP) were added, and the reaction mixture was stirred in an inert argon atmosphere overnight, and then it was decomposed with 17% aq. hydrochloric acid [14,18]. Layers were separated, and the aqueous layer was extracted with ethyl acetate (2 × 20 mL). The combined organic solution was washed with brine (10 mL) and dried with anhydrous magnesium sulphate. The solvent was removed, and the crude white product was purified by column chromatography (eluent toluene/*tert*-butyl methyl ether, 30/1, *v*/*v*). To a cold (0 °C) solution of aldehyde in acetone, Jones reagent was added dropwise under stirring. The reaction mixture was left to heat up to room temperature and stirred overnight. Then, the reaction mixture was poured onto crushed ice (400 mL). After all the ice had melted, the formed precipitate was filtered, washed with cold water and dried in air. The crude product was purified via crystallisation from hexane.

#### 2.1.2. Method (B)

A mixture of acid **1a** with a catalytic amount of *N*,*N*-dimethylformamide (DMF) (0.05 mL) in oxalyl chloride (10–20 mL) was stirred at room temperature overnight. The unreacted oxalyl chloride was distilled off, and the residue was boiled for 1 min in hexane with a spatula-tip of active carbon. The suspension was filtered while hot and the solvent was evaporated. The crude acid chloride was dissolved in dry DCM (3 mL) and added dropwise to a cold (0 °C) solution of 4-hydroxybenzoic acid and DMAP in dry dichloromethane. The mixture was stirred at 0 °C in the argon atmosphere for 10 h. The reaction was then decomposed with 17% aq. hydrochloric acid. Layers were separated, and the aqueous layer was extracted with chloroform (2 × 20 mL). The combined organic solution was washed with brine (10 mL) and dried with anhydrous magnesium sulphate. The solvent was removed, and the crude white product was purified by column chromatography.

#### 2.1.3. Method (C) 

A mixture of acid **1a** with a catalytic amount of DMF (0.05 mL) in oxalyl chloride (10–20 mL) was stirred under reflux for 2 h [17]. Unreacted oxalyl chloride was distilled off, and the crude product was boiled for 1 min in hexane with a spatula-tip of active carbon. The suspension was filtered while hot and the solvent was evaporated. The crude acid chloride was dissolved in dry dichloromethane (3 mL) and added dropwise to a solution of 4-hydroxybenzoic acid and DMAP in dry DCM. The mixture was stirred and heated under reflux in the argon atmosphere for 5 h. Then, it was cooled to the ambient temperature and decomposed with 17% aq. hydrochloric acid. Layers were separated, and the aqueous layer was extracted with chloroform (2 × 20 mL). The combined organic solution was washed with brine (10 mL) and dried with anhydrous magnesium sulphate. The solvent was removed, and the crude white product was purified by column chromatography.

### 2.2. Synthesis of the Target Liquid Crystal

**Figure 2:**
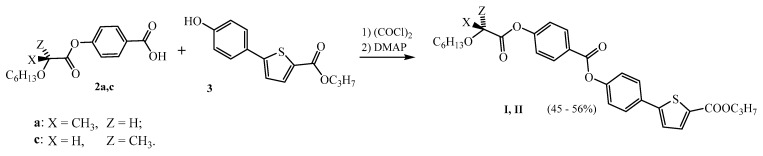
Synthesis of target LCs.

The synthesis of the target liquid crystalline material (Figure 2) started from chiral acids **2a** and **2c** prepared according to methods (A) and (B), respectively, which provided the best results. The acids were first transformed to appropriate acid chlorides, which were subsequently used for the acylation of hydroxy ester **3**, prepared according to a method described in the literature [19], in the presence of DMAP as a base.

### 2.3. Chiral HPLC Separation of Chiral Building Blocks

**Figure 3:**
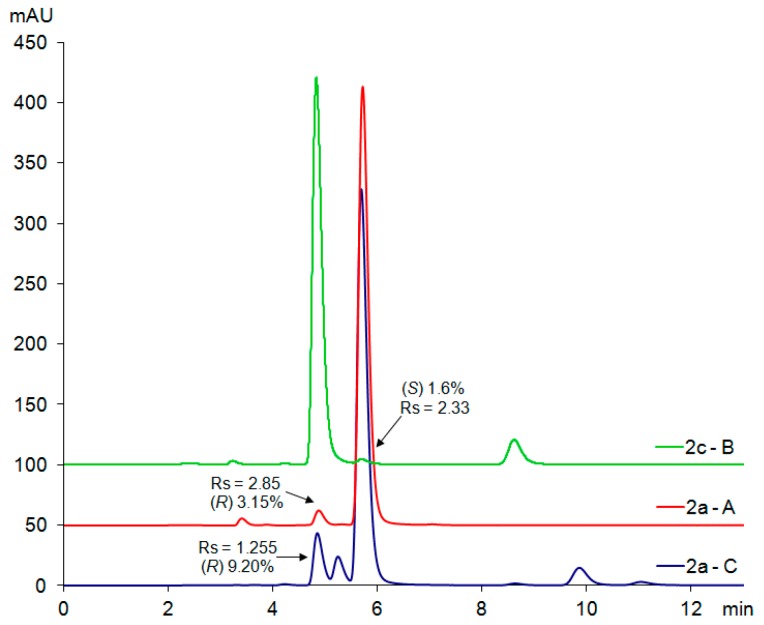
Enantioseparation of chiral acids **2a** and **2c** prepared according to experimental methods A–C in HPLC mode using ECOM HPLC system.

The chiral separation method was based on a previously optimised methodology available in our laboratory for the enantioseparation of chiral photosensitive LCs [11,12,13]. Optimal separation conditions for precursors **2a**–**d** were heptane/propan-2-ol (9/1, *v*/*v*). Pure (*R*)- and (*S*)-enantiomers were screened individually, and the position of the enantiomeric impurity in the main substance was determined by comparing the retention times (Figure 3).

Under the given conditions, baseline resolution of the enantiomers was feasible. In addition to that, it was possible to study the effect of the particular synthetic strategy on enantiomeric purity of the chiral building blocks. The results (Table 1) show that synthetic method A provided the chiral building block **2a** with *ee* = 93.7%. The chemical purity of the substance was high. Synthetic method B, which was first used for the preparation of **2c**, gave rise to a product with higher optical purity (*ee* = 96.8%) than method A. However, several other chemical impurities were detected. An attempt to improve the reaction rate by heating up the reaction mixture (method C [17]) provided the target acid **2a** containing a broad range of chemical impurities, as well as the opposite enantiomer **2c**, resulting in *ee* = 81.6%. Similar results were obtained for acids **2b** and **2d**, possessing the C12 terminal alkyl chain (see Figure S1). Therefore, it is clear that method C should not be used for the synthesis of chiral building blocks **2a**–**d** or compounds possessing a similar structure with lactic acid as the chiral unit.

Since the optical purity of starting esters, as stated by the manufacturer, is *ee* = 98% for (*S*)-ethyl lactate and *ee* = 99% for (*R*)-methyl lactate, partial racemisation also occurred when using the synthetic methods A and B. First, we decided to modify method A, because it is well known that DCC (and other carbodiimides) may induce partial or full racemisation of a chiral acid during its activation [20,21]. Therefore, O-(1H-6-chlorobenzotriazol-1-yl)-1,1,3,3-tetramethyluronium tetrafluoroborate (TCTU), a more selective coupling reagent, was employed for the synthesis of **2a** using a method described for analogous reagents [22]. However, an almost identical enantiomeric composition of **2a** as for DCC-mediated reaction was achieved (see Figure S2).

The results indicate that the partial racemisation observed for building blocks prepared according to methods A and B most probably takes place during the first two steps of the synthesis, although the methodology was previously described as racemisation-free [8,23]. Therefore, it is reasonable to assume that with the development of new analytical approaches, the precision of impurity determination has been significantly improved. The current technology of chiral CSPs obviously beats the former methods based on optical rotation measurements and determination of enantiomeric excess by transformation of the respective chiral building blocks to an amide [8].

### 2.4. Chiral HPLC Separation of Full-Length Liquid-Crystalline Materials

**Figure 4:**
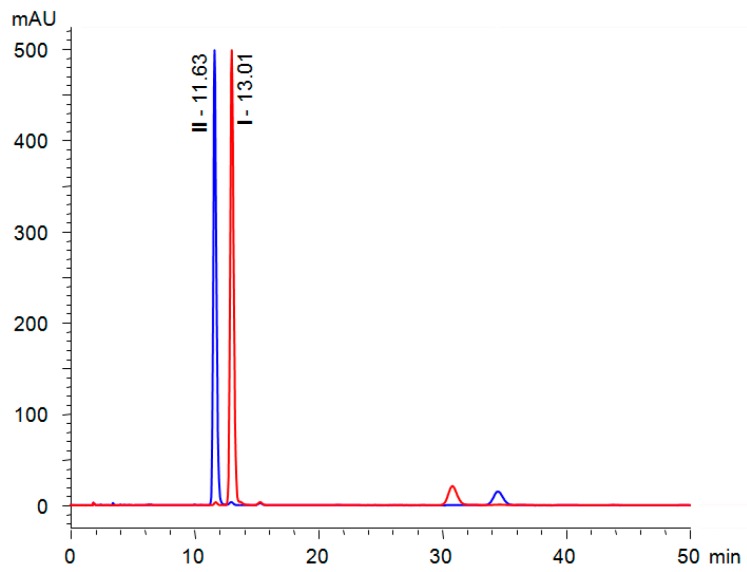
Chiral separation of target chiral liquid crystals. Overlaid chromatograms of **I** (red trace) and **II** (blue trace) acquired with Chiralpak AD-3 using Acquity UPC2 operated in HPLC mode; mobile phase heptane/IPA (9/1, *v*/*v*), flow rate 1 mL/min, temperature 25 °C, sample concentration 0.5 mg/mL, injection volume 5 µL.

The developed separation method for the precursors was further successfully applied for the chiral separation of full-length liquid crystals (R_S_ = 2.5). Due to the shift in the absorption maximum of the target compound, the detection wavelength was set to 308 nm. To determine contamination of target CLCs with an opposite enantiomer, we prepared corresponding (*S*)- and (*R*)-enantiomers (**I** and **II**, respectively), and analysed them independently (Figure 4).

Analogously to the chiral precursors, (*R*)-enantiomer **II** eluted first. This documents that the enantiorecognition of the enantiomers is strictly governed by the substituents near the chiral centre, while the rest of the molecule only contributes to retention. Interestingly, the analysis of (*S*)-enantiomer **I** revealed *ee* = 98.8% and for target material **II** ((*R*)-enantiomer) *ee* = 98.4% was found. This finding contradicts the results obtained for the chiral precursors, which showed higher optical purity of the (*R*)-enantiomer. We can speculate that the target CLCs could be enantiomerically enriched via crystallisation, leading, in consequence, to target materials with higher optical purity than the starting compounds. However, intensive purification will be used, because the initial application of column chromatography and three consecutive crystallisation still provided a rather impure material (Figure 4).

### 2.5. Chiral SFC and SFC-MS Separation of Full-Length Liquid-Crystalline Materials

**Figure 5:**
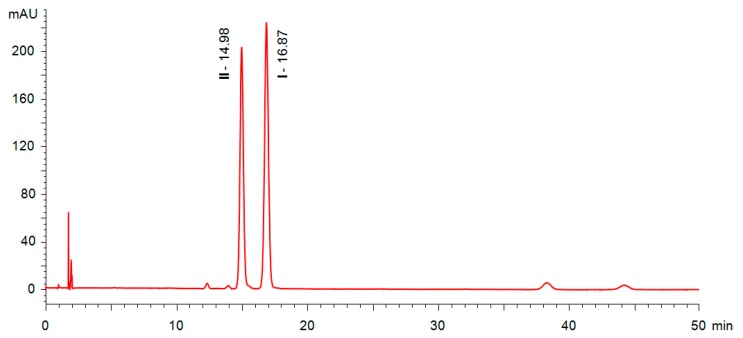
Mixture of **I** and **II** analysed in SFC mode on Chiralpak AD-3 to directly compare the performance of the column in HPLC and SFC mode. Mobile phase scCO_2_/IPA (70/30), sample concentration 0.5 mg/mL, injection volume 5 µL, temperature 30 °C, backpressure 2000 psi and the flow rate of 1 mL/min were used.

Recently, it has been shown that SFC offers striking selectivity in the enantioseparation of chiral liquid crystals [9,10]. Therefore, we prepared a spiked sample, which enabled us to determine the elution order of enantiomers and match the chiral impurities (observed in HPLC mode) to the corresponding materials. Indeed, the SFC analysis provided better resolution (R_S_ = 3.1), not only for the target materials, but also for chiral impurities present in the materials (Figure 5). The elution order of **I** and **II** remained unchanged; material **II** eluted in the first peak.

It should be noted that the SFC separation was, in fact, performed outside the supercritical region of the fluid [24,25]. Yet, the term SFC is generally also accepted for the subcritical fluid region (also subFC), because the main advantages of lower viscosity and higher diffusivity of the mobile phase are fully preserved [26]. Unlike normal phase HPLC, direct coupling to a mass spectrometer (detector) is easily accessible in SFC. Therefore, we took this advantage to control the optical purity of the materials and also to shed light on their impurity profile. Due to specific SFC conditions, a dedicated SFC column (ChiralArt Amylose-C 250 × 4.6 mm, i.d., 5 µm) was used in all following SFC measurements. It should be noted that a dedicated SFC column should be used for each chromatographic mode, because a frequent change of chromatographic modes could reduce column lifetime. Although it is natural to use short columns with small particles in SFC (due to low viscosity and higher diffusivity of the fluid), in some cases it is economic to use older type of columns for SFC method development, in particular, when unusual conditions (e.g., outside supercritical region, highly acidic mobile phase) are expected to be used.

**Figure 6:**
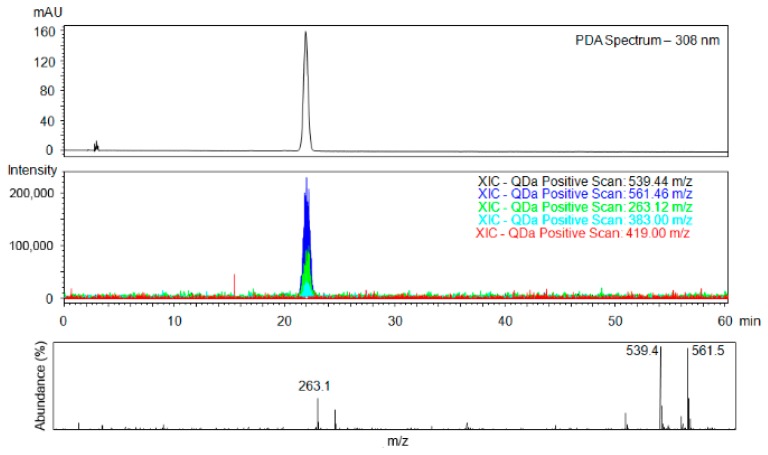
Analysis of purified target material **I** performed in SFC-MS mode. Upper part shows the UV trace, lower part shows reconstructed chromatograms (XIC) with the mass of the target material 539.4 [M + H]^+^, 561.5 [M + Na]^+^, fragments 263.1 [M + H]^+^, 383.0 [M + H]^+^, and the main impurity 419.0 [M + H]^+^ and mass spectrum of the product. Conditions: ChiralArt Amylose-C (250 × 4.6 mm, i.d., 5 µm) column, mobile phase scCO_2_/IPA (70/30), flow rate of 1 mL/min, sample concentration 0.5 mg/mL, injection volume 5 µL, temperature 30 °C, backpressure 2000 psi, ESI+.

Analysis of the target material **I** (Figure 6) clearly demonstrates that the additional purification with gradient elution column chromatography and subsequent multiple crystallisations provide the chemically and enantiomerically pure material. No signals of impurities have been detected, vide infra. Furthermore, only a negligibly increased baseline was observed in the area where the peak of (*R*)-enantiomer (material **II**) should be present. Since the limit of quantification for SFC-MS is below 1 µg/mL (for details see ESI), the enantiomeric purity of the target material **I** analysed under the given conditions (Figure 6) is *ee* > 99.6%.

**Figure 7:**
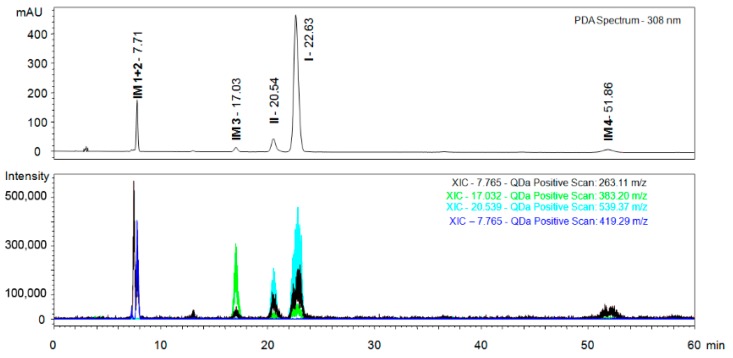
SFC-MS analysis of major impurities (IM) of the target compound **I**. Upper part shows the UV trace, lower part reconstructed chromatograms with masses of the target materials and impurities originating from the synthesis. Conditions: ChiralArt Amylose-C (250 × 4.6 mm, i.d., 5 µm) column, mobile phase scCO_2_/IPA (70/30), flow rate of 1 mL/min, sample concentration 1.0 mg/mL, injection volume 10 µL, temperature 30 °C, backpressure 2000 psi, ESI+.

For the analysis of impurities, impurity fractions of material **I** obtained from the column chromatography and mother liquor after re-crystallisation were collected and used. Since the mobile phase composed of scCO_2_ and IPA (low molar mass alcohols in general) is acidic [27,28], ESI+ was the preferred mode also for the impurities analysis. Apart from the target material **I** and the enantiomeric impurity (material **II**), several other substances were identified (Figure 7). The SFC-MS analysis shows that molecular masses of impurities correspond to fragments observed for ionised target materials. This documents that all impurities present in the target material originate from the starting compounds and side reactions among them—mainly migration of *O*-alkyllactic acid from the target materials to phenol **3** (for more detailed analysis and structures of the impurities see ESI in the Supplementary Materials).

## 3. Materials and Methods

### 3.1. Chemicals

Heptane, dichloromethane (DCM), methanol (MeOH), and propan-2-ol (IPA) were purchased from LachNer s.r.o. (Neratovice, Czech Republic); all solvents were of HPLC grade. Carbon dioxide (SFC grade) was obtained from Linde Industrial Gasses (Prague, Czech Republic). Chemicals used for the synthesis of the chiral precursors and target LCs were commercial products from Sigma-Aldrich (Prague, Czech Republic), Fischer Scientific (Pardubice, Czech Republic) and they were used without further purification. Silica gel (Kieselgel 60) for the purification of the intermediates and target LCs was purchased from Merck (Darmstadt, Germany).

### 3.2. Instrumentation and Methods

The optical purity of chiral precursors was determined by HPLC using Chiralpak^®^ AD-3 (150 × 4.6 mm ID, 3 μm) from Chiral Technologies Europe (Illkirch, France) column in a heptane/IPA (9/1) mixture, the flow rate was set to 1 mL/min, temperature 25 °C. Sample concentration was 0.2 mg/mL, injection volume 20 µL and detection wavelength was set to 235 nm. Optical purity of final liquid crystalline materials was verified under the same conditions, except for the detection wavelength, which was set to 305 nm. The ECOM HPLC system consisted of Alpha pump (ECOM, Prague, Czech Republic), CT050 controller (AZ Chrom, Bratislava, Slovakia) and ECDA2000 (ECOM, Prague, Czech Republic) detector. A part of HPLC measurements on Chiralpak AD-3 was carried out on an SFC system (operated in the HPLC mode), as specified below.

SFC measurements were performed on a SFC-dedicated column, namely ChiralArt Amylose-C (250 × 4.6 mm ID, 5 µm) from YMC Europe GmbH (Dinslaken, Germany) using an Acquity Ultra-Performance Convergence Chromatography (UPC2) system equipped with a binary solvent manager, sample manager, convergence manager, column manager 30S for eight 250 mm columns and PDA detector and single quadrupole mass detector (QDa) from Waters (Milford, CT, USA). The mobile phase consisted of supercritical carbon dioxide (scCO_2_) with 30% of IPA. The flow rate was set to 1 mL/min, injection volume to 5 μL. For all measurements, the backpressure was set to 2000 psi (138 bar) and the column temperature to 30 °C. The sample concentration was 0.5 mg/mL in a mixture of heptane/IPA (9/1, *v*/*v*). The void volume (*t*0) was determined from the first negative peak observed after the injection. An equilibration window of 15 min was applied prior the first sample injection for each column. The PDA acquisition range was 210–400 nm and the detection wavelength was 308 nm. The mass detection was performed in positive ion mode (ESI+) with a mass range 200.00–600.00 Da, cone voltage 10 V. The ESI spray voltage was set to 0.8 kV. Empower 3 software was used for system control and data acquisition.

Nuclear magnetic resonance (NMR) spectra were acquired using an Agilent 400-MR DDR2 spectrometer (Santa Clara, CA, USA) operating at 400.13 MHz for ^1^H and 100.62 MHz for ^13^C cores. Elemental analysis was performed on a Perkin-Elmer 2400 Instrument (Waltham, MA, USA).

### 3.3. Experimental

#### (*S*)-Propyl 5-{4-(4-(2-hexyloxypropanoyloxy)benzoyloxy)phenyl}thiophene-2-carboxylate (**I**)

A mixture of acid **2a** (500 mg, 1.71 mmol) with a catalytic amount of DMF (0.05 mL) in oxalyl chloride (20 mL) was stirred at reflux for 2 h. The excess of the oxalyl chloride was distilled off and the residue was heated under reflux in hexane with a spatula-tip of active carbon for 1 min. The suspension was filtered, and the solvent evaporated; the crude acid chloride was dissolved in dry dichloromethane and added dropwise to a solution of hydroxyl ester **3** (374 mg, 1.43 mmol) with DMAP (174 mg, 1.43 mmol) in dry dichloromethane (25 mL). The reaction mixture was stirred in argon atmosphere at room temperature for 2 h. Then, the reaction mixture was decomposed with 17% aq. hydrochloric acid, layers were separated, and the aqueous layer was extracted with toluene. The combined organic solution was washed with brine and dried with anhydrous magnesium sulphate. The solvent was removed, and the crude product was purified by column chromatography (toluene/*tert*-butyl methyl ether 20:1, *v*/*v*) and multiple crystallisations from an ethyl acetate/ethanol mixture to obtain 430 mg (56%) of a white solid.

It should be noted that the purification procedure given above was found to be insufficient (vide infra), and therefore both target materials **I** and **II** were further purified. This additional purification step consisted of column chromatography using gradient elution with CHCl_3_ – 0.8% MeOH in CHCl_3_ and subsequent crystallisation from ethanol. The gradient elution column chromatography afforded a chemically pure substance while the subsequent crystallisation served as a tool for removal of the trace amount of the opposite enantiomer, which was confirmed by SFC-MS measurements.

^1^H-NMR (400 MHz, CDCl_3_): 0.89 (t, 3H, CH_3_), 1.02 (t, 3H, CH_3_), 1.23–1.45 (m, 8H, CH_2_), 1.60 (d, 3H, CH_3_), 1.79 (m, 2H, CH_2_), 3.52 (m, 1H, OCH_2_), 3.69 (m, 1H, OCH_2_), 4.22 (q, 1H, OCH), 4.28 (t, 2H, OCH_2_), 7.26–7.30 (m, 5H, H_ar_), 7.70 (d, 2H, *J* = 8.7 Hz, H_ar_), 7.77 (d, 1H, *J* = 3.9 Hz, H 21), 8.26 (d, 2H, *J* = 8.8 Hz, H_ar_). ^13^C-NMR (100 MHz, CDCl_3_): 10.46 (CH_3_), 14.04 (CH_3_), 18.67 (CH_3_), 22.13 (CH_2_), 22.59 (CH_2_), 25.72 (CH_2_), 29.73 (CH_2_), 31.62 (CH_2_), 66.75 (OCH_2_), 70.85 (OCH_2_), 74.97 (OCH), 121.74 (CH_ar_), 122.40 (CH_ar_), 123.84 (CH_ar_), 126.93 (C), 127.40 (CH_ar_), 131.45 (C), 131.90 (CH_ar_), 132.79 (C), 134.22 (CH_ar_), 149.81 (C), 151.12 (C), 154.78 (C), 162.27 (C), 164.15 (C), 171.46 (C). Elemental analysis for C_30_H_34_O_7_S (538.67), calculated C 66.89, H 6.36, S 5.95%, found C 67.02, H 6.44, S 5.86%.

In the similar way, starting from acid **2c**, (*R*)-propyl 5-{4-(4-( 2-hexyloxypropanoyloxy)benzoyloxy)phenyl}thiophene-2-carboxylate (**II**) was prepared, 300 mg (45%). ^1^H-NMR: (400 MHz, CDCl_3_): 0.89 (t, 3H, CH_3_), 1.03 (t, 3H, CH_3_), 1.24–1.45 (m, 8H, CH_2_), 1.59 (d, 3H, CH_3_), 1.79 (m, 2H, CH_2_), 3.52 (m, 1H, OCH_2_), 3.69 (m, 1H, OCH_2_), 4.22 (q, 1H, OCH), 4.27 (t, 2H, OCH_2_), 7.26–7.30 (m, 5H, H_ar_), 7.69 (d, 2H, *J* = 8.7 Hz, H_ar_), 7.77 (d, 1H, *J* = 3.9 Hz, H 21), 8.26 (d, 2H, *J* = 8.8 Hz, H_ar_). ^13^C-NMR (100 MHz, CDCl_3_): 10.46 (CH_3_), 14.04 (CH_3_), 18.67 (CH_3_), 22.13 (CH_2_), 22.59 (CH_2_), 25.72 (CH_2_), 29.73 (CH_2_), 31.62 (CH_2_), 66.75 (OCH_2_), 70.85 (OCH_2_), 74.97 (OCH), 121.74 (CH_ar_), 122.40 (CH_ar_), 123.84 (CH_ar_), 126.93 (C), 127.40 (CH_ar_), 131.45 (C), 131.90 (CH_ar_), 132.79 (C), 134.22 (CH_ar_), 149.81 (C), 151.12 (C), 154.78 (C), 162.27 (C), 164.15 (C), 171.46 (C). Elemental analysis for C_30_H_34_O_7_S (538.67), calculated C 66.89, H 6.36, S 5.95%, found C 66.54, H 6.37, S 5.92%.

## 4. Conclusions

In this study, we focused on the evaluation of optical purity of lactic acid-based building blocks used for the synthesis of a broad range of CLCs (including the target LCs). We have shown that frequently utilised synthetic procedures do not afford enantiomerically pure building blocks. The use of such compounds does not inevitably result in impure target materials; however, very precise control of chemical and optical purity must be employed. Partial racemisation occurring during the synthesis of CLCs could result in modification of mesomorphic properties of the target materials. This may have caused the discrepancies in the mesomorphic behaviour of the same CLCs reported by different research groups. Most importantly, slight modification of enantiomeric composition of the chiral material could potentially lead to malfunctioning of a CLC-based device. Therefore, precise contemporary analytical methods with low limits of detection should be used to secure required quality of CLCs used in research, development and applications.

## Figures and Tables

**Table 1:** Relative peak areas of chiral building blocks **2a** and **2c** obtained from chiral HPLC analysis and corresponding enantiomeric excess values of the respective enantiomers.

Peak Area (%)	Method A	Method B	Method C
**2a**	96.85	1.60	90.80
**2c**	3.15	98.40	9.20
**% *ee***	93.70	96.80	81.60

## Supplementary Materials

The supplementary information is available online.
