# Supplementary material for: Determination of Optical Purity of Lactic Acid-Based Chiral Liquid Crystals and Corresponding Building Blocks by Chiral High-Performance Liquid Chromatography and Supercritical Fluid Chromatography
Source: Molecules. 2019 Mar 20;24(6):1099. doi: 10.3390/molecules24061099 (PMC6471377; doi:10.3390/molecules24061099)
Supplement: Supplementary file 1 [file molecules-24-01099-s001.pdf]

Determination of optical purity of lactic acid-based chiral liquid crystals and their building blocks by chiral high performance liquid chromatography and supercritical fluid chromatography

Anna Poryvai,[1] Terezia Vojtylová-Jurkovičová,[2] Michal Šmahel,[1] Natalie Kolderová,[3] Petra Tomášková,[2] David Sýkora,[4] Michal Kohout[1,]*

This electronic supplementary file provides analytical data of *O*-alkyl lactic acids. Supporting data on chiral separation of chiral building blocks, enantiomeric purity of these compounds obtained by different synthetic methods, limits of detection and quantification for the target materials as well as their SFC-MS impurity profile are also provided.

1. Characterization of intermediates 1a-d

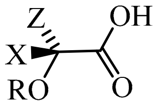

1a-d

**1a**	$R = C_6H_{13}$,	$X = CH_3$,	$Z = H$;
**1b**	$R = C_{12}H_{25}$,	$X = CH_3$,	$Z = H$;
**1c**	$R = C_6H_{13}$,	$X = H$,	$Z = CH_3$,
**1d**	$R = C_{12}H_{25}$,	$X = H$,	$Z = CH_3$.

Scheme S-1. Structure and designation of chiral units used for the synthesis of target chiral liquid crystals.

(S)-2-(Hexyloxy)propanoic acid (1a)

^{1}H NMR (400 MHz, $CDCl_3$): 0.88 (t, 3H, CH_3), 1.25-1.36 (m, 6H, 3×CH_2), 1.45 (d, 3H, CH_3, J = 7.0 Hz), 1.61 (m, 2H, $OCH_2\underline{CH_2}$), 3.54 (m, 2H, OCH_2), 4.00 (m, 1H, OCH). ^{13}C NMR (100 MHz, $CDCl_3$): 14.0 (CH_3), 18.10 (CH_3), 22.6 (CH_2), 25.63 (CH_2), 29.6 (CH_2), 31.6 (CH_2), 70.6 (OCH), 74.5 (OCH_2), 177.1 (COOH).

(S)-2-(Dodecyloxy)propanoic acid (1b)

^{1}H NMR (400 MHz, $CDCl_3$): 0.88 (t, 3H, CH_3), 1.26-1.34 (m, 18H, 9×CH_2), 1.45 (d, 3H, CH_3, J = 7.2 Hz), 1.62 (m, 2H, $OCH_2\underline{CH_2}$), 3.54 (m, 2H, OCH_2), 3.99 (m, 1H, OCH). ^{13}C NMR (100 MHz, $CDCl_3$): 14.3 (CH_3), 18.34 (CH_3), 22.8 (CH_2), 26.1 (CH_2), 29.5-29.8 (7×CH_2), 32.1 (CH_2), 70.8 (OCH), 74.7 (OCH_2), 177.8 (COOH).

(R)-2-(Hexyloxy)propanoic acid (1c)

^{1}H NMR (400 MHz, $CDCl_3$): 0.88 (t, 3H, CH_3), 1.25-1.37 (m, 6H, 3×CH_2), 1.45 (d, 3H, CH_3, J = 7.0 Hz), 1.61 (m, 2H, $OCH_2\underline{CH_2}$), 3.54 (m, 2H, OCH_2), 4.00 (m, 1H, OCH). ^{13}C NMR (100 MHz, $CDCl_3$): 14.0 (CH_3), 18.1 (CH_3), 22.6 (CH_2), 25.6 (CH_2), 29.6 (CH_2), 31.6 (CH_2), 70.6 (OCH), 74.5 (OCH_2), 177.1 (COOH).

(R)-2-(Dodecyloxy)propanoic acid (1d)

^{1}H NMR (400 MHz, $CDCl_3$): 0.88 (t, 3H, CH_3), 1.26-1.36 (m, 18H, 9×CH_2), 1.45 (d, 3H, CH_3, J = 7.0 Hz), 1.61 (m, 2H, $OCH_2\underline{CH_2}$), 3.54 (m, 2H, OCH_2), 3.99 (m, 1H, OCH). ^{13}C NMR (100 MHz, $CDCl_3$): 14.3 (CH_3), 18.4 (CH_3), 22.8 (CH_2), 26.1 (CH_2), 29.5-29.8 (7×CH_2), 32.1 (CH_2), 70.8 (OCH), 74.7 (OCH_2), 177.8 (COOH).

Similarly to acids **1a**-**1d**, analytical data of chiral building blocks **2a**-**2d** comply with data published in the literature.[S1, S2] For readers' reference, the characterization of materials obtained with synthetic Method A are given below.

(S)-4-(2-(Hexyloxy)propanoyloxy)benzoic acid (2a)

^{1}H NMR (300 MHz, $CDCl_3$): 0.89 (t, 3H, CH_3), 1.28-1.41 (m, 6H, 3×CH_2), 1.57-1.70 (m, 5H, H_{alk}), 3.51 (m, 1H, OCH_2), 3.70 (m, 1H, OCH_2), 4.22 (m, 1H, CH), 7.23 (d, 2H, J = 8.8 Hz), 8.17 (d, 2H, J = 8.8 Hz). ^{13}C NMR (100 MHz, $CDCl_3$): 14.1 (CH_3), 18.4 (CH_3), 22.6 (CH_2), 25.9 (CH_2), 29.8 (CH_2), 31.7 (CH_2), 70.8 (CH_2), 75.1 (CH), 121.5 (CH_{ar}), 127.2 (C_{ar}), 131.9 (CH_{ar}), 154.6 (C_{ar}), 171.4 (C=O), 171.6 (C=O).

(S)-4-(2-(Dodecyloxy)propanoyloxy)benzoic acid (2b)

^{1}H NMR (400 MHz, $CDCl_3$): 0.87 (t, 3H, CH_3), 1.25-1.41 (m, 18H, 9×CH_2), 1.57-1.69 (m, 5H, H_{alk}), 3.51 (m, 1H, OCH_2), 3.70 (m, 1H, OCH_2), 4.22 (m, 1H, CH), 7.23 (d, 2H, J = 8.6 Hz), 8.17 (d, 2H, J = 8.6 Hz). ^{13}C NMR (100 MHz, $CDCl_3$): 14.2 (CH_3), 18.8 (CH_3), 22.8 (CH_2), 26.2 (CH_2), 29.5-29.9 (7×CH_2), 32.1 (CH_2), 71.2 (CH_2), 75.4 (CH), 122.3 (CH_{ar}), 127.7 (C_{ar}), 132.7 (CH_{ar}), 155.7 (C_{ar}), 172.2 (C=O), 172.4 (C=O).

(R)-4-(2-(Hexyloxy)propanoyloxy)benzoic acid (2c)

^{1}H NMR: 0.88 (t, 3H, CH_3), 1.,25-1.37 (m, 18H, 3×CH_2), 1.57-1.70 (m, 5H, CH_2, CH_3), 3.49 (m, 1H, OCH_2), 3.68 (m, 1H, OCH_2), 4.20 (m, 1H, CH), 7.23 (d, 2H, J = 8.8 Hz), 8.14 (d, 2H, J = 8.8 Hz). ^{13}C NMR (100 MHz, $CDCl_3$): 14.0 (CH_3), 18.6 (CH_3), 22.6 (CH_2), 25.7 (CH_2), 29.7 (CH_2), 31.6 (CH_2), 70.8 (CH_2), 74.9 (CH), 121.5 (CH_{ar}), 127.0 (C_{ar}), 131.9 (CH_{ar}), 154.8 (C_{ar}), 171.2 (C=O), 171.5 (C=O).

(S)-4-(2-(Dodecyloxy)propanoyloxy)benzoic acid (2d)

^{1}H NMR: 0.88 (t, 3H, CH_3), 1.25-1.37 (m, 18H, 9×CH_2), 1.57-1.70 (m, 5H, CH_2, CH_3), 3.49 (m, 1H, OCH_2), 3.68 (m, 1H, OCH_2), 4.20 (m, 1H, CH), 7.23 (d, 2H, J = 8.8 Hz), 8.14 (d, 2H, J = 8.8 Hz). ^{13}C NMR (100 MHz, $CDCl_3$): 14.2 (CH_3), 18.8 (CH_3), 22.7 (CH_2), 26.2 (CH_2), 29.5-30.1 (7×CH_2), 32.2 (CH_2), 71.2 (CH_2), 75.4 (CH), 122.3 (CH_{ar}), 127.9 (C), 132.7 (CH_{ar}), 155.7 (Car), 172.0 (C=O), 172.3 (C=O).

2. Chiral separation of building blocks

It should be noted that the structure of the chiral building blocks plays an important role in chiral separation. Increasing lipophilicity of the substances with increasing length of the alkyl chains from C6 to C12 led to shortening of retention times (less preferred adsorption on the stationary phase due to better solubility in the mobile phase). Moreover, with increasing length of the terminal alkyl chain, the resolution drops significantly. Nevertheless, for the non-racemic mixture of **2b** and **2d**, satisfactory enantioseparation was observed; the elution order was the same as for **2a** and **2c**, *i.e.* (*R*)- before (*S*)-enantiomer. However, when analysing enantiomeric purity of **2d**, it is reasonable to assume that the minor (*S*)-enantiomer may be covered by tailing of the major peak. Modification of the mobile phase composition did not provide increased resolution and the reversal of elution order by using a different CSP was not pursued.

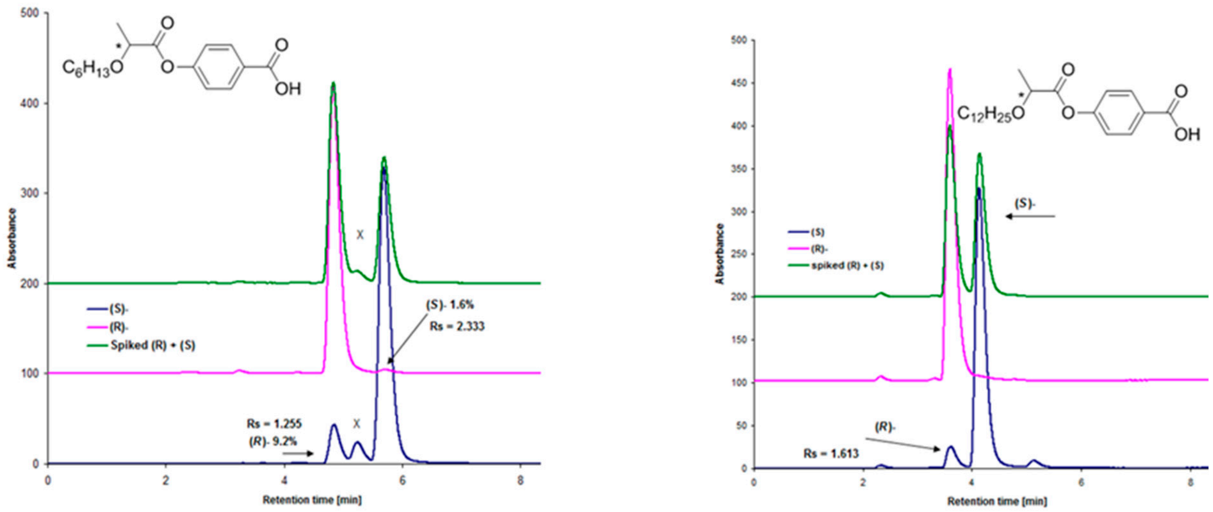

Figure S-1. Comparison of enantioseparation of **2a** and **2c** (left) with that of **2b** and **2d** (right) on Chiralpak® AD-3 (150×4.6 mm i.d., 4.6 µm) using ECOM HPLC in a heptane/IPA (9/1, v/v) mixture at the flow rate of 1 mL/min and temperature 25 °C. Sample concentration was 0.2 mg/mL, injection volume 20 µL and detection wavelength was set to 235 nm. Only synthetic methods (B) and (C) are shown.

Following chromatograms show the similarity in the purity of acid **2a** prepared according to two different coupling agents with the aim to avoid possible DCC-induced racemisation.

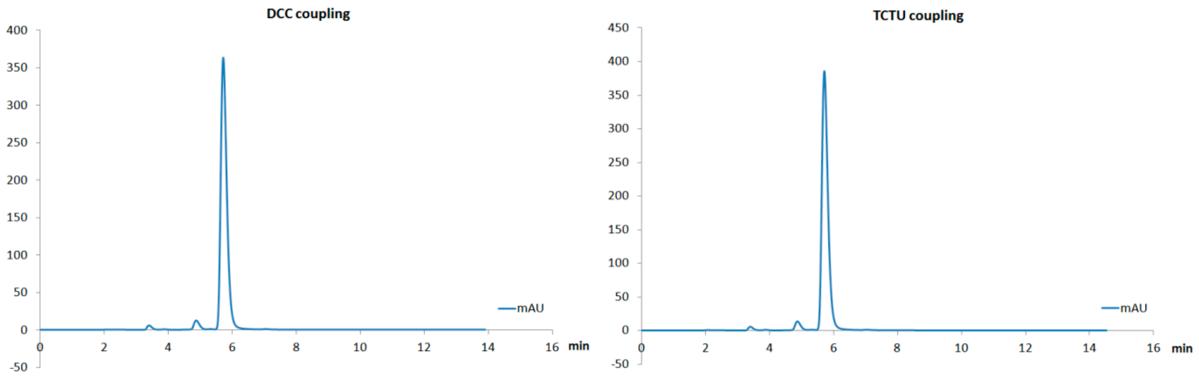

Figure S-2. Enantiomeric purity control of building blocks, prepared using two different coupling reagents, performed on Chiralpak AD-3 using ECOM HPLC; mobile phase heptane/propan-2-ol (9/1, v/v), flow rate 1 mL/min, temperature 25 °C, sample concentration 0.2 mg/mL, injection volume 20 µL.

3. Limit of detection and limit of quantification

In the SFC mode, the limits were measured using a series of calibration solutions (Table S-1). The signal-to-noise (S/N) ratio was calculated automatically by the Empower software according to European pharmacopoeia standard. For two consecutive measurements of a calibration curve, the limit of quantification for the SFC method with PDA detection is approximately 10 µg/mL and the limit of detection is found between 2.5 µg/mL and 5 µg/mL. The linearity of the method in the given interval has also been verified, the respective plot for the first measurements is shown in **Figure S-3**.

Table S-1.
Analysis of calibration solutions of the target material **I** using PDA detection, the first measurement is depicted on the left side, the second measurement on the right side of the table.

c (μg/mL)	t (min)	area (μV*sec)	S/N	c (μg/mL)	t (min)	area (μV*sec)	S/N
50	23.141	706500	52.1	50	23.155	703644	50.3
25	23.139	351346	26.1	25	23.164	356110	26.3
10	23.139	150695	11.4	10	23.165	145578	11.0
7.5	23.142	107775	8.2	7.5	23.153	102267	7.4
5	23.166	73173	5.3	5	23.155	72715	5.5
2.5	23.178	31049	2.8	2.5	23.159	36299	2.9
1	23.030	12093	1.2	1	23.159	13812	1.3

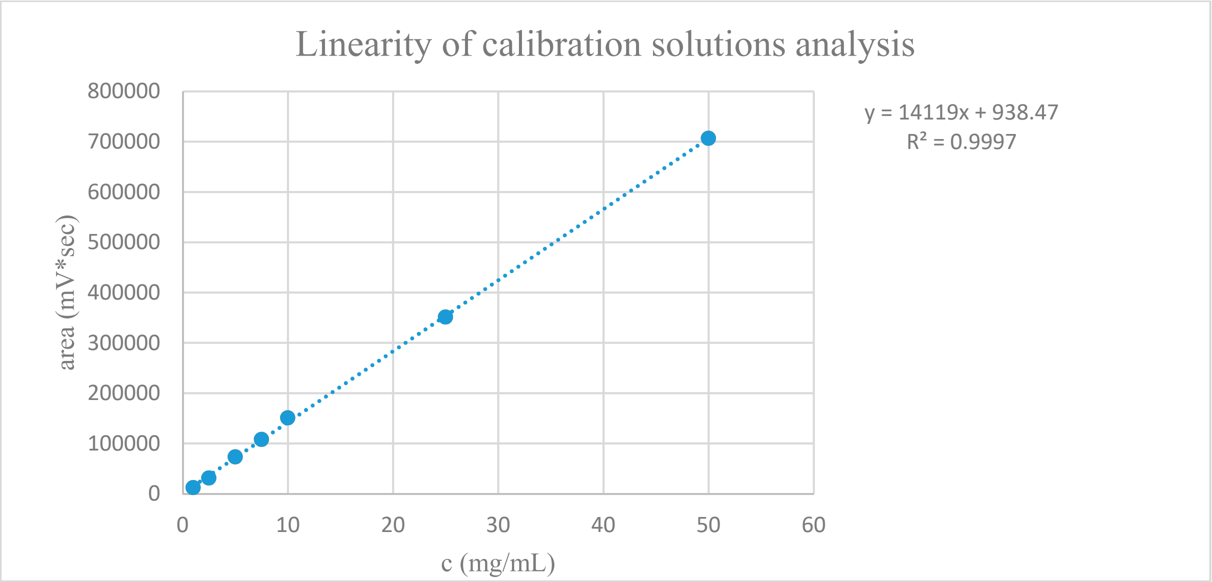

Figure S-3. Linear regression for the first measurements of calibration solutions.

For the current SFC-MS method, the limit of detection and quantification was not determined. This is due to the fact that the method has not been fully optimized in terms of mass detector parameters, such as gas flow, probe temperature, ionisation potential. The current MS method provides about one order of magnitude higher sensitivity than the PDA detection, which is sufficient for the present study.

4. Impurity profiling of target materials

Impurities present in the target materials that correspond to the masses of fragments obtained upon their ionisation can be easily located in the spectrum.

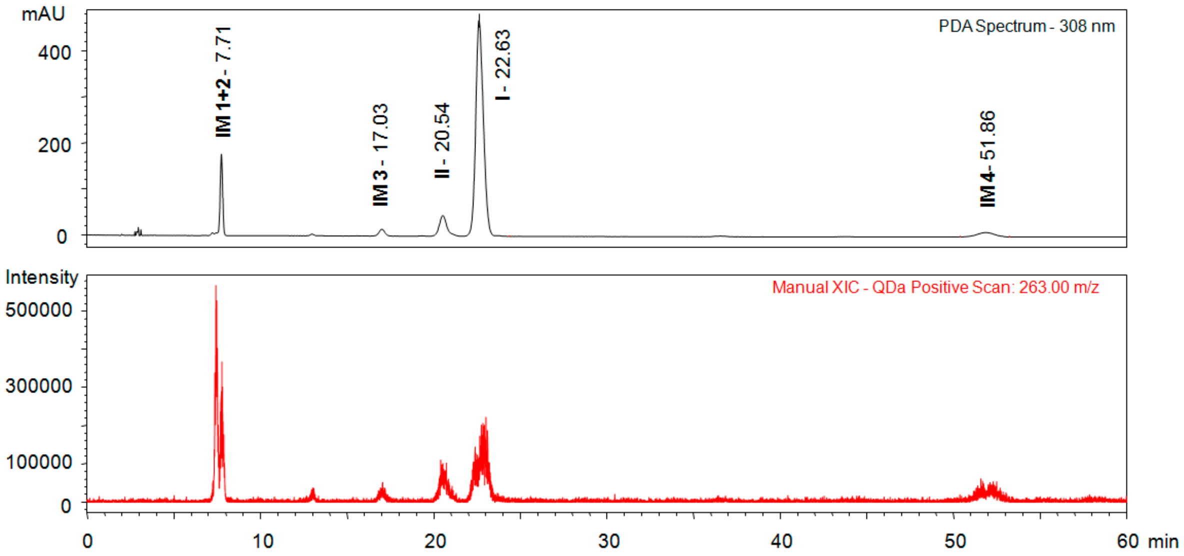

Figure S-4. Identification of an impurity located at 7.71 min. Conditions: ChiralArt Amylose-C (250×4.6 mm, i.d., 5 µm) column, mobile phase scCO_2/IPA (70/30), flow rate of 1 mL/min, sample concentration 1.0 mg/mL, injection volume 10 µL, temperature 30 °C, backpressure 2000 psi, ESI+.

It is assumed that the peak at 7.71 min in PDA actually shows two compounds due to the presence of small fronting part of the major peak (**Figure S-4**). This assumption has been confirmed by SFC-MS analysis, where the mass $[M+H]^+$ 263 Da was found in the front part of the peak and with smaller intensity also in the main body of the peak. Therefore it seems that the compound present in the front part (small shoulder in PDA) of the impurity peak is the starting hydroxy ester **3**, which ionizes well under the applied conditions. On the other hand, the second impurity present in the major part of the peak present in PDA chromatogram at 7.71 min ionized rather badly under the applied conditions (**Figure S-5**). The mass $[M+H]^+$ 419.3 Da seems to be the correct mass of the second impurity. This mass corresponds to a substance generated by a side reaction via migration of the *O*-hexyllactic acid unit to hydroxy ester **3** (**Figure S-6**). Such migration of lactic acid unit has already been observed in our laboratory and products of such migration have already been isolated preparatively (data not shown). Therefore, it is assumed that the structure is correctly assigned.

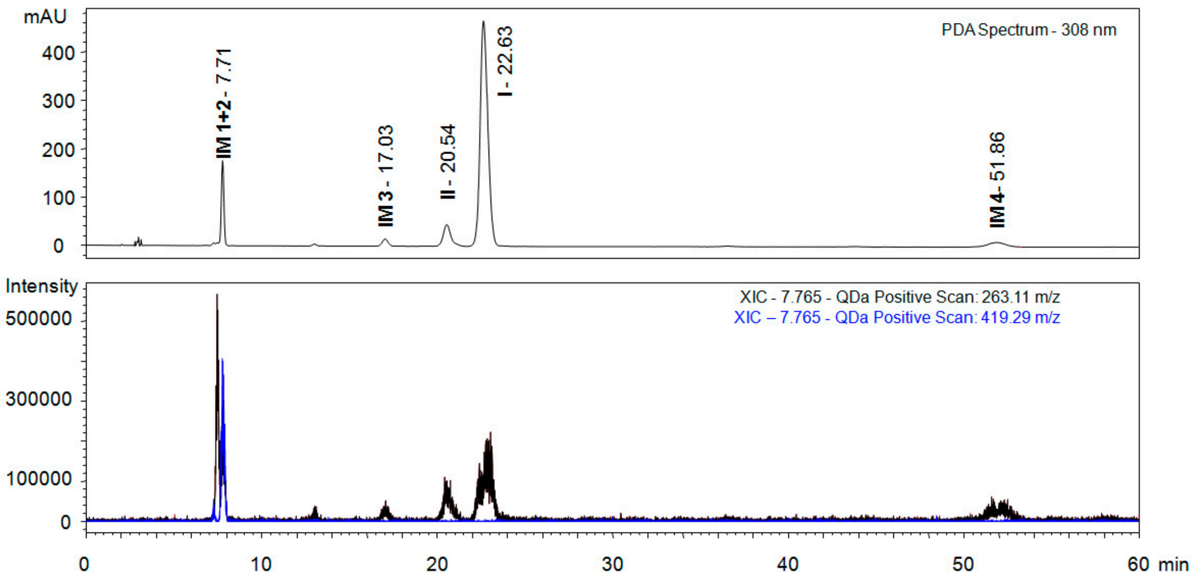

Figure S-5. Identification of the second impurity located at 7.71 min. Conditions: ChiralArt Amylose-C (250×4.6 mm, i.d., 5 µm) column, mobile phase $scCO_2$/IPA (70/30), flow rate of 1 mL/min, sample concentration 1.0 mg/mL, injection volume 10 µL, temperature 30 °C, backpressure 2000 psi, ESI+.

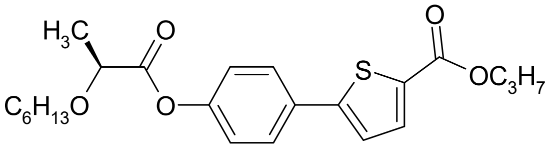

Figure S-6. The structure of the second impurity located at 7.77 min.

Furthermore, we could identify the second part of the target material formed by the migration of the lactic acid unit – corresponding hydroxy ester possessing three aromatic cores (**Figure S-7**). This substance should have mass of $[M+H]^+$ 383.0 Da and, indeed, such compound has been detected among the isolated impurities (**Figure S-7**).

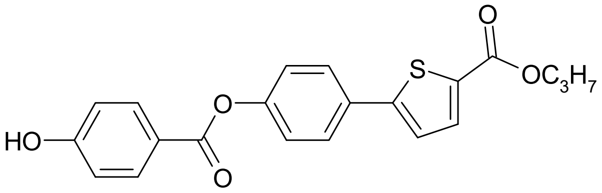

Figure S-7. The structure of the impurity located at 17.03 min.

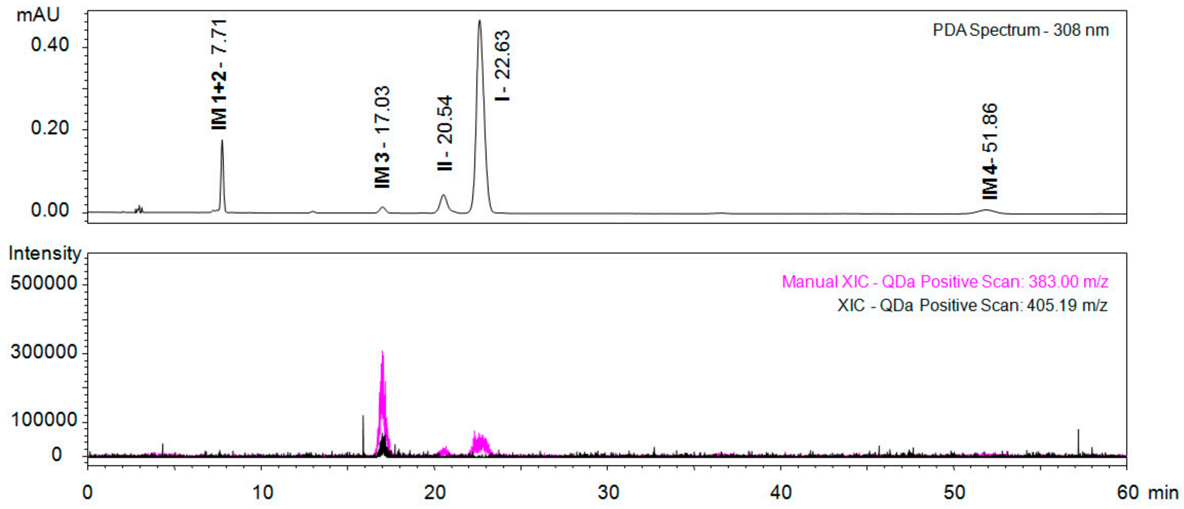

Figure S-8. Identification of the third impurity located at 17.03 min, $[M+H]^+$ 383.0 Da and 405.2 $[M+Na]^+$. Conditions: ChiralArt Amylose-C (250×4.6 mm, i.d., 5 µm) column, mobile phase $scCO_2$/IPA (70/30), flow rate of 1 mL/min, sample concentration 1.0 mg/mL, injection volume 10 µL, temperature 30 °C, backpressure 2000 psi, ESI+.

Apart from these well identified impurities, another chiral substance should be present. Since, we were not able to detect this chiral substance, clearly visible in HPLC-UV and SFC-UV (IM 4 at 51.86 min), it is assumed that this substance is an acid (**Figure S-9**). Due to the structure of this acid, it is assumed that it would not ionize under the given SFC-MS ESI+ conditions.

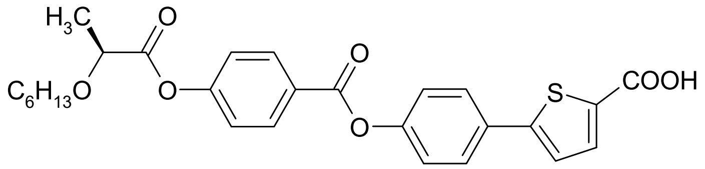

Figure S-9. The structure of the assumed chiral impurity located at 51.86 for material **I**.

Reference

[S1] Kohout, M.; Bubnov, A.; Šturala, J.; Novotná, V.; Svoboda, J. Effect of alkyl chain length in the terminal ester group on mesomorphic properties of new chiral lactic acid derivatives. *Liq. Cryst.* **2016**, *43*, 1472-1485.

[S2] Černovská, K.; Košata, B.; Svoboda, J.; Novotná, V.; Glogarová, M. Novel ferroelectric liquid crystals based on fused thieno[3,2-b]furan and thieno[3,2-b]thiophene cores. *Liq. Cryst.* **2006**, *33*, 987-996.
